# Evolution of Multiple Domains of the HIV-1 Envelope Glycoprotein during Coreceptor Switch with CCR5 Antagonist Therapy

**DOI:** 10.1128/spectrum.00725-22

**Published:** 2022-06-21

**Authors:** Yueqi Du, Ellen Wu, Xiang Gao, Jie Zhang, John C. Martin, Bruce A. Rosa, Makedonka Mitreva, Lee Ratner

**Affiliations:** a Division of Oncology, Washington University School of Medicine, St. Louis, Missouri, USA; b Division of Infectious Diseases, Washington University School of Medicine, St. Louis, Missouri, USA; c McDonnell Genome Institute, Washington University School of Medicine, St. Louis, Missouri, USA; National Institutes of Health

**Keywords:** HIV, Envelope, CCR5, CXCR4, Coreceptor, CD4, Vicriviroc, HIV

## Abstract

HIV-1 uses CD4 as a receptor and chemokine receptors CCR5 and/or CXCR4 as coreceptors. CCR5 antagonists are a class of antiretrovirals used to inhibit viral entry. Phenotypic prediction algorithms such as Geno2Pheno are used to assess CCR5 antagonist eligibility, for which the V3 region is screened. However, there exist scenarios where the algorithm cannot give an accurate prediction of tropism. The current study examined coreceptor shift of HIV-1 from CCR5-tropic strains to CXCR4-tropic or dual-tropic strains among five subjects in a clinical trial of the CCR5 antagonist vicriviroc. Envelope gene amplicon libraries were constructed and subjected to next-generation sequencing, as well as single-clone sequencing and functional analyses. Approximately half of the amplified full-length single envelope-encoding clones had no significant activity for infection of cells expressing high levels of CD4 and CCR5 or CXCR4. Functional analysis of 9 to 21 individual infectious clones at baseline and at the time of VF were used to construct phylogenetic trees and sequence alignments. These studies confirmed that specific residues and the overall charge of the V3 loop were the major determinants of coreceptor use, in addition to specific residues in other domains of the envelope protein in V1/V2, V4, C3, and C4 domains that may be important for coreceptor shift. These results provide greater insight into the viral genetic determinants of coreceptor shift.

**IMPORTANCE** This study is novel in combining single-genome sequence analysis and next-generation sequencing to characterize HIV-1 quasispecies. The work highlights the importance of mutants present at frequencies of 1% or less in development of drug resistance. This study highlights a critical role of specific amino acid substitutions outside V3 that contribute to coreceptor shift as well as important roles of the V1/V2, V4, C3, and C4 domain residues.

## INTRODUCTION

HIV-1 is the cause of a global infection of more than 75 million individuals over the last 4 decades (1). Effective vaccines and curative therapies remain to be developed (2, 3). One of the major challenges in prevention and treatment efforts is the high rate of genetic divergence of HIV-1 strains (4). This results from the error-prone nature of the reverse transcriptase, with 0.2 errors per genome during each replication cycle (5). Additional errors occur during transcription by RNA polymerase II. In addition, HIV-1 replication is dynamic, with a viral generation time of 2.5 days with 1,010 to 1,012 new virions produced each day in an infected individual (6). Frequent recombination and natural selection further enhance the rate of evolutionary change (7). This has resulted in multiple different clades of HIV-1 associated with different geographic areas (4). The persistent nature of HIV infection results in evolution both within and among hosts (8).

One of the most striking findings about HIV-1 genetic diversity is the extensive variation in the envelope gene (*env*), particularly sequences encoding the hypervariable domains (V1 to V5) (9). The viral envelope is a trimeric complex of the 120-kDa surface glycoprotein (SU, gp120), noncovalently associated with the gp41 transmembrane protein (TM) (10). The envelope protein mediates virus binding and entry (11). Therefore, it is the major target of neutralizing antibodies (12). Virus entry is initiated by SU binding to the primary receptor, CD4 (11). This leads to a conformational change, with alterations in the V1/V2 loops allowing exposure of the V3 loop, which binds to cellular coreceptors (13–15). The principal coreceptors for HIV-1 are C-C chemokine receptor 5 (CCR5) and C-X-C chemokine receptor 4 (CXCR4) (13). Most infections of patients occur with HIV-1 strains that utilize CCR5, which are termed R5-tropic strains (16, 17). However, over time, sequence changes in the envelope protein occur that allow the use of CXCR4 (18). These strains are designated X4-tropic if they utilize CXCR4 and not CCR5, or dual- or R5X4-tropic if they can utilize either coreceptor (19). After coreceptor binding, additional conformational changes occur that open the trimeric envelope complex and lead to a conformational change in the TM protein, which inserts into the host membrane and promotes virus-cell membrane fusion (20).

The emergence of HIV-1 drug resistance remains one of the greatest challenges in treatment (8). Latent HIV-1 reservoirs in patients on highly active antiretroviral therapy serve to replenish the pool of replicating virus from a variety of CD4^+^ cell types in tissues throughout the body (21). In addition to the approved reverse transcriptase, integrase, and protease inhibitors, there has been considerable effort to develop therapies that block HIV-1 entry (22). Maraviroc (MVC) was developed as an inhibitor of envelope binding to CCR5 (23). However, its specificity for CCR5, rather than CXCR4, restricted its use to individuals with only R5-tropic virus. Although several genotypic algorithms were developed, such as the Gene2Pheno prediction tool, they were not sufficiently accurate for clinical use (24). Thus, phenotypic assays, such as the Trofile assay, are required to accurately identify coreceptor use (25). Other inhibitors of HIV-1 entry have been developed to block CD4 binding or TM conformational changes, but these drugs require parenteral administration or are restricted to use for individuals that have failed multiple other treatment regimens (22, 26, 27).

The current work examines isolates obtained from subjects on a trial of an alternate CCR5 antagonist, vicriviroc (VCV) (28). Although VCV has similar activity to MVC, the pharmaceutical producer chose not to continue its therapeutic development (29). The inhibitory spectrum of VCV appears very similar to that of MVC (30, 31). Thus, we further analyzed the samples from a phase II clinical trial of vicriviroc, derived from the subset of individuals who developed virological failure (VF) and a shift from CCR5 to CXCR4 use while on treatment (28).

Eligibility for this trial was based on the presence in the plasma of exclusively R5 virus, as determined by the Trofile tropism assay, and VF while receiving a ritonavir-containing regimen, with a plasma HIV-1 level of at least 5,000 copies/mL. Eight subjects who experienced protocol-defined VF were selected out of a total of nine subjects with VF and change in coreceptor use from the entire population of 106 protocol subjects (28).

Samples were obtained at two time points: study baseline (time point 1) and VF (time point 2) (32). The amount of time between time points 1 and 2 varied between subjects, ranging from 2 to 32 weeks. *env* sequence libraries were generated under limiting dilution conditions for each subject (33) and cloned into the pNL4-3Env-Luc+ plasmid containing an infectious molecular clone of HIV-1 lacking the *env* gene, with the firefly luciferase gene in place of the *nef* gene. Illumina sequences were generated for each amplicon library. A clustering analysis using single-nucleotide polymorphism (SNP) information of the 16 amplicon libraries was performed (34, 35). Analysis of the V3 coding sequence showed that there was a similar degree of heterogeneity in each library (33). Moreover, the clustering pattern demonstrated that V3 sequences from the two time points for each subject were more similar to one another than to those of other subjects, in agreement with a previous study (33, 36).

In earlier studies of subject 1, the amplicon libraries were passed through U87.CD4.CCR5 and U87.CD4.CXCR4 cell lines to select functional libraries (33). The U87 cell line is a human glioma cell line with no significant expression of CCR5 or CXCR4. U87 cells were stably transfected with human CD4 and human CCR5 or CXCR4 cDNAs to express high levels of each corresponding protein (37). *env* sequences selected on the individual U87 cell lines from subject 1 were characterized by next-generation sequencing (NGS), and differences were identified for each time point (33).

In an early study of subjects 2 and 5, a portion of quasispecies was found to retain the R5-tropic phenotype at VF. To investigate their drug resistance, individual infectious molecular clones from amplicon libraries from each time point were isolated, sequenced, and characterized for functional activity on U87.CD4.CCR5 and U87.CD4.CXCR4 cell lines (33, 38). For time point 1, all molecular clones from both subjects were found to be R5-tropic. At time point 2, 42% of functional clones from subject 2 and 98% of functional clones from subject 5 were R5-tropic (38). Phylogenetic trees suggested that the V3 domain as well as the V1/V2 domains contributed to the VCV-resistant phenotype of the R5-tropic clones. The contribution of the V1/V2 domains to drug resistance was validated by swapping these domains between sensitive and resistant clones (38).

In the current study, of subjects 1, 3, 4, 6, 7, and 8, who demonstrated HIV shift primarily to X4-using clones after VCV treatment, we further assessed the mechanism of VCV resistance. Functional analysis of individual clones obtained at baseline and after development of VF and also sequence and phylogenetic analyses were employed to identify the mechanistic basis for VF and coreceptor shift.

## RESULTS

### Panel of recombinant *env* clones.

**FIG 1:**
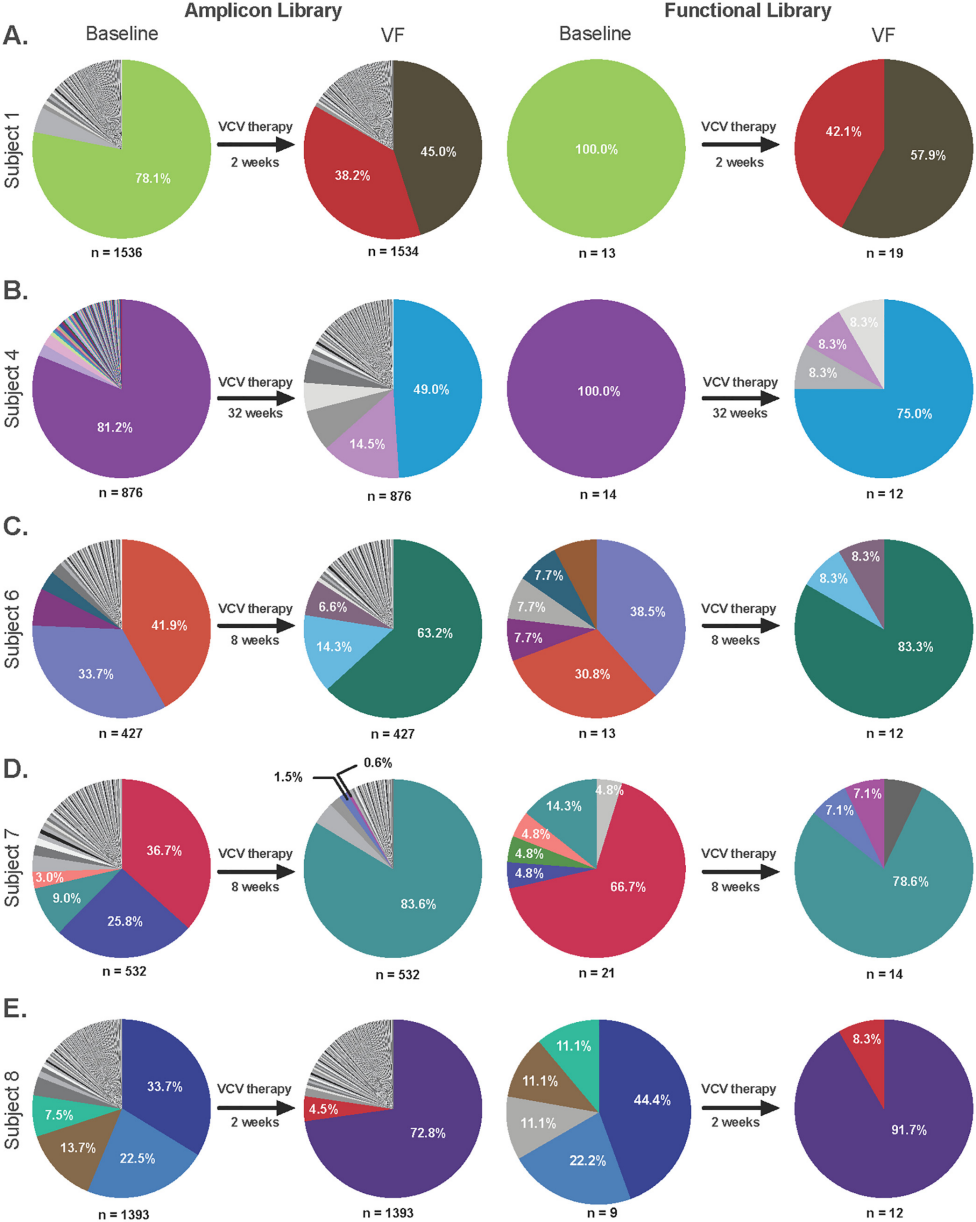
V3 Sequence analyses of amplicon and functional *env* libraries. Amplicon libraries were generated for each of the 5 subjects at baseline and at the time of VF. The duration of VCV therapy prior to VF is shown in each case. NGS results are shown for each amplicon library, with the number of complete sequences in each case indicated below the pie graph. Each slice of the pie has a unique color for a specific clone, and the percentages of clones with identical V3 sequences are indicated. Up to 50 individual single-amplicon clones were selected for functional analysis in infection assays. The proportion of identical V3 sequences for each of 9 to 21 functional clones for each time point are indicated, using the same color code as used for the amplicon library pie graphs.

Samples from subjects 1, 3, 4, 6, 7, and 8, which shifted primarily to X4-using clones after VCV treatment, were utilized in the current study. Individual molecular clones from time points 1 and 2 of subjects 1, 4, 6, 7, and 8 were isolated, sequenced, and characterized for coreceptor use. An insufficient number of clones from subject 3 were obtained for these analyses, and subject 3 was excluded from the study. Analysis of V3 sequences by the Geno2Pheno algorithm confirmed that the majority of clones switched from R5-tropic to X4-using after treatment with VCV. The exception was subject 7, from whom 9.0% of baseline clones were predicted to be X4-using clones. Results of NGS analysis of the V3 sequences in the amplicon libraries are shown in Fig. 1. Below each pie graph are shown the number of reads obtained with full-length V3 domain sequences. The slice of each pie denotes the proportion of each sequence variant found within the library, with identical colors used to indicate identical V3 sequences. The most predominant clone at baseline accounted for 33.7 to 81.2% of sequences. For subjects 1 and 4, a single predominant clone was found, but for subjects 6 to 8, additional subdominant clones were detected, accounting for 3.0 to 33.7% of the population.

V3 sequences for VF samples were obtained from time point 2, after 2 to 32 weeks of VCV therapy (Fig. 1). Predominant clones represented 45.0 to 83.6% of the population, and subdominant clones accounted for 0.6 to 38.2% of sequences. It is notable that V3 sequences at time point 2 were found in only a small proportion of sequences from time point 1, if at all. In contrast, subject 7 showed significant expansion of preexisting clones under VCV therapy. To prevent the expansion of preexisting dual-tropic clones, patients must undergo a phenotypic assay prior to starting CCR5-antagonist therapy. Subject 7 is a classic example of what happens when the Trofile assay fails to accurately capture the quasispecies tropism. The most common X4-using clone expanded from 9.0% to 83.6% following treatment. These results highlight the need for accurate and sensitive screening of tropism prior to starting CCR5-antagonist therapy. Combining both phenotypic and genotypic methods provides a better representation of a patient’s quasispecies.

The functional libraries represent those selected individual clones from each time point that were capable of infecting U87.CD4.CCR5 or U87.CD4.CXCR4 cells at levels significantly above the background level. Sequences were obtained from 9 to 21 functional clones from baseline time point 1 and 12 to 19 functional clones from the VF time point 2 from each of the 5 subjects (Fig. 1). Although some differences can be seen in the proportion of each V3 sequence obtained at each time point in the functional library compared to the amplicon library, these differences were not statistically significant (*P* > 0.99).

### Functional analyses of infectious molecular clones.

**FIG 2:**
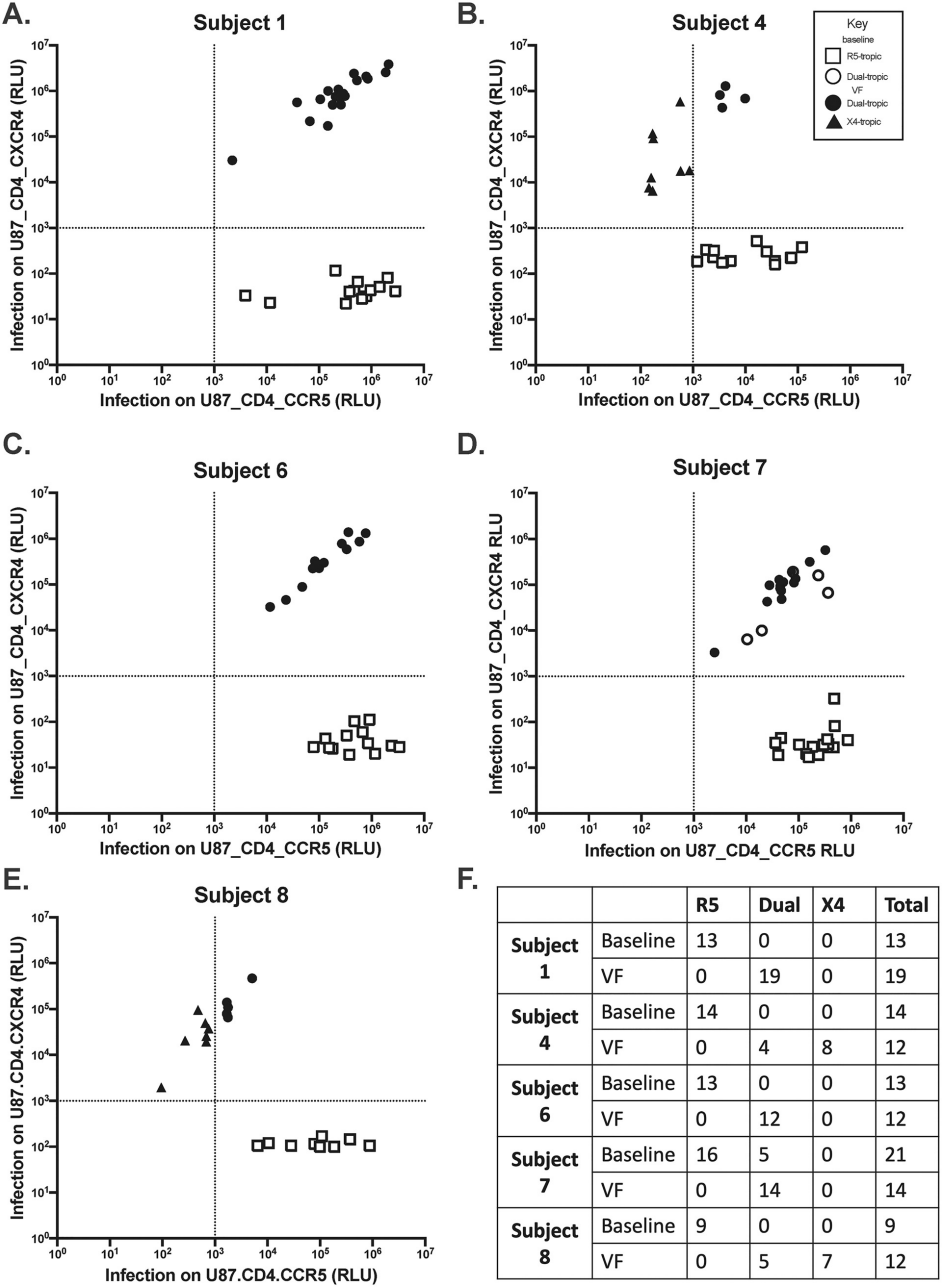
Activity of functional envelope clones of each of the five subjects for infection of U87.CD4 cells expressing CCR5 or CXCR4. (A to E) Tropism was determined by a single-round infection assay. Recombinant viral constructs were transfected into HEK 293T cells to produce replication-competent virions. Viral supernatants were used to infect CCR5- and CXCR4-expressing U87.CD4 cell lines. Clones with an average luciferase readout of >2 standard deviations above the average value for negative-control wells were defined as positive for coreceptor usage. RLU values were normalized such that the cutoff was 1,000 RLU. Nonfunctional clones are not shown. RLU obtained 24 h after infection of clones obtained at baseline (open symbols) and at VF (closed symbols) are shown in panels A to E for each subject, with squares for R5 clones, circles for dual-tropic clones, and triangles for X4 clones. (F) Summary table of the number of functional clones of each type obtained for each subject at baseline and the time of VF. All assays were performed at least twice.

Results of the functional analyses are shown in Fig. 2. Levels of luciferase activity varied from 10^3^ to 10^7^ relative light units (RLU) among clones. It is notable that approximately half of the clones from each time point were nonfunctional, i.e., levels of infection were not significantly different from background levels on both U87.CD4.CCR5 and U87.CD4.CXCR4 cell lines (data not shown).

All baseline clones (open symbols) were R5-tropic (Fig. 2, squares), with the exception of subject 7, for whom 5 of 21 baseline clones were dual-tropic clones (Fig. 2, circles). For VF time point 2, 33.3 to 100% of clones were dual-tropic (Fig. 2, filled circles) and 0 to 67.3% were X4-tropic (filled triangles). X4-tropic clones were only detected at time point 2 in subjects 4 and 8.

### Genetic differences between baseline and VF clones.

**FIG 3:**
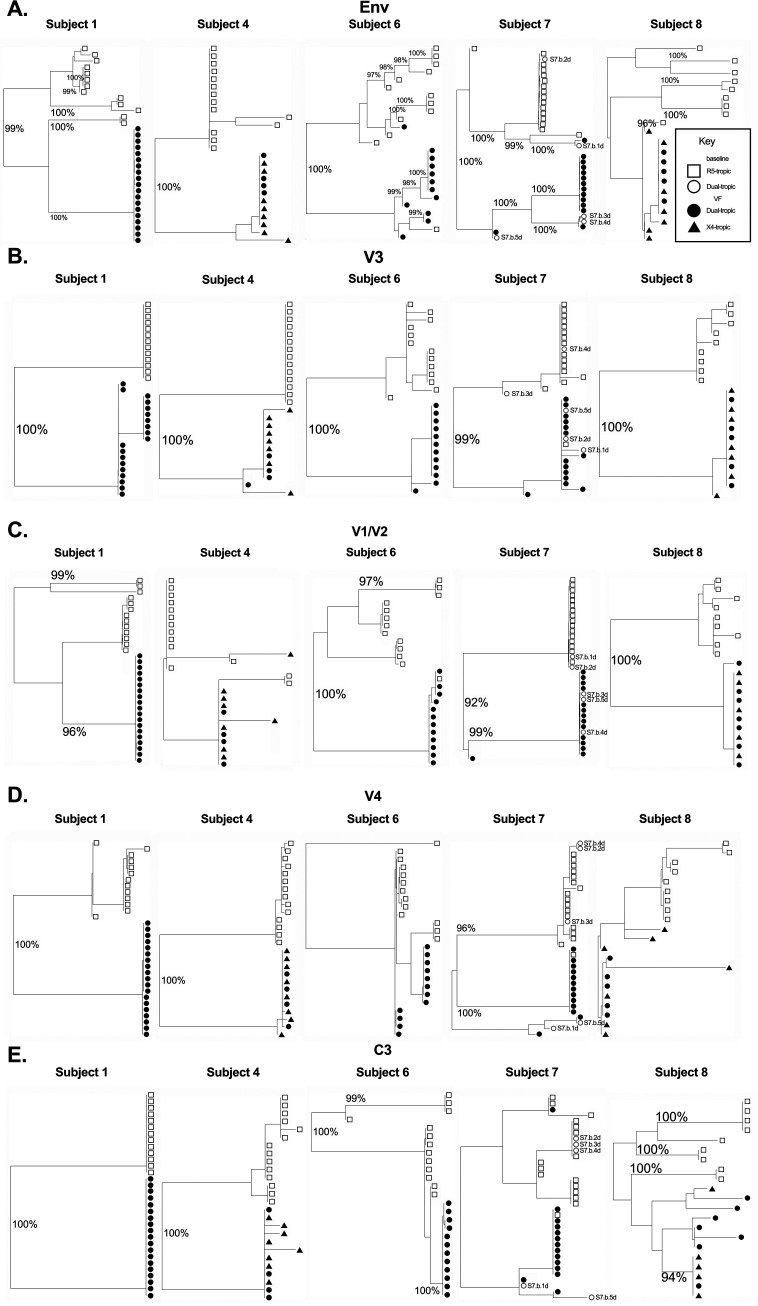
Phylogenetic trees of the full-length envelope protein (A), V3 loop (B), V1/V2 loops (C), V4 loop (D), and C3 (E). Results of the hierarchical analysis of variants from functional libraries based on full-length amino acid sequences are shown for each subject. Branch points with bootstrap values ≥95% are labeled. The symbols are identical to those used in Fig. 2, with open symbols for baseline clones, closed symbols for those obtained at VF, squares for R5 clones, circles for dual-tropic clones, and triangles for X4 clones. Designations for specific outlier clones are also provided.

Phylogenetic analyses of full-length Env and of each variable and constant region were performed to identify genetic signatures of envelope amino acid sequence evolution. Phylogenetic trees for the full-length envelope sequence are shown in Fig. 3A, utilizing the same symbols as in Fig. 2. In all five subjects, sequences from before and after VCV treatment were found on significantly different branches, with the exception of a small number of baseline sequences which clustered with VF sequences for subjects 6 and 7. One VF sequence from each of subjects 6 and 7 was found to cluster with baseline sequences. Although R5 and non-R5 sequences were on separate branches with the exception of one sequence each from subject 6 and subject 7, X4-tropic and dual-tropic sequences were not found on separate branches in subjects 4 and 8, the only subjects in which X4-tropic sequences were identified.

Phylogenetic analysis of the V3 amino acid sequence showed that sequences clustered into two groups for all 5 subjects (Fig. 3B). Statistically significant differences, based on bootstrap assays, were detected between baseline (open symbols) and VF sequences (closed symbols). Three outlier, preresistant clones were detected in subject 7 (S7.b.3d, S7.b.4d, S7.b.5d). Significant clustering was also seen for V3 sequences, based on tropism, with R5-tropic clones clustered separately from non-R5-tropic clones. No significant differences were seen between V3 sequences of X4 and dual-tropic clones (subjects 4 and 8). Similar clustering is seen in phylogenetic trees for the V1/V2, V4, and C3 domains (Fig. 3C to E).

**FIG 4:**
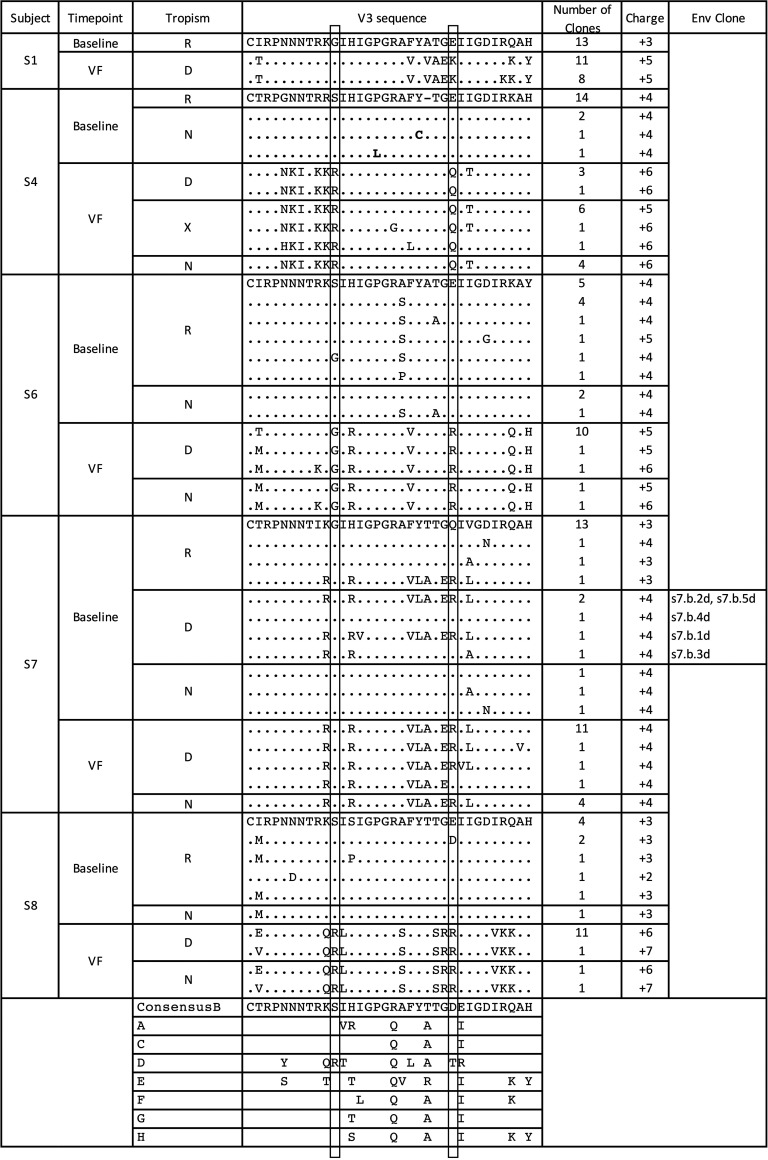

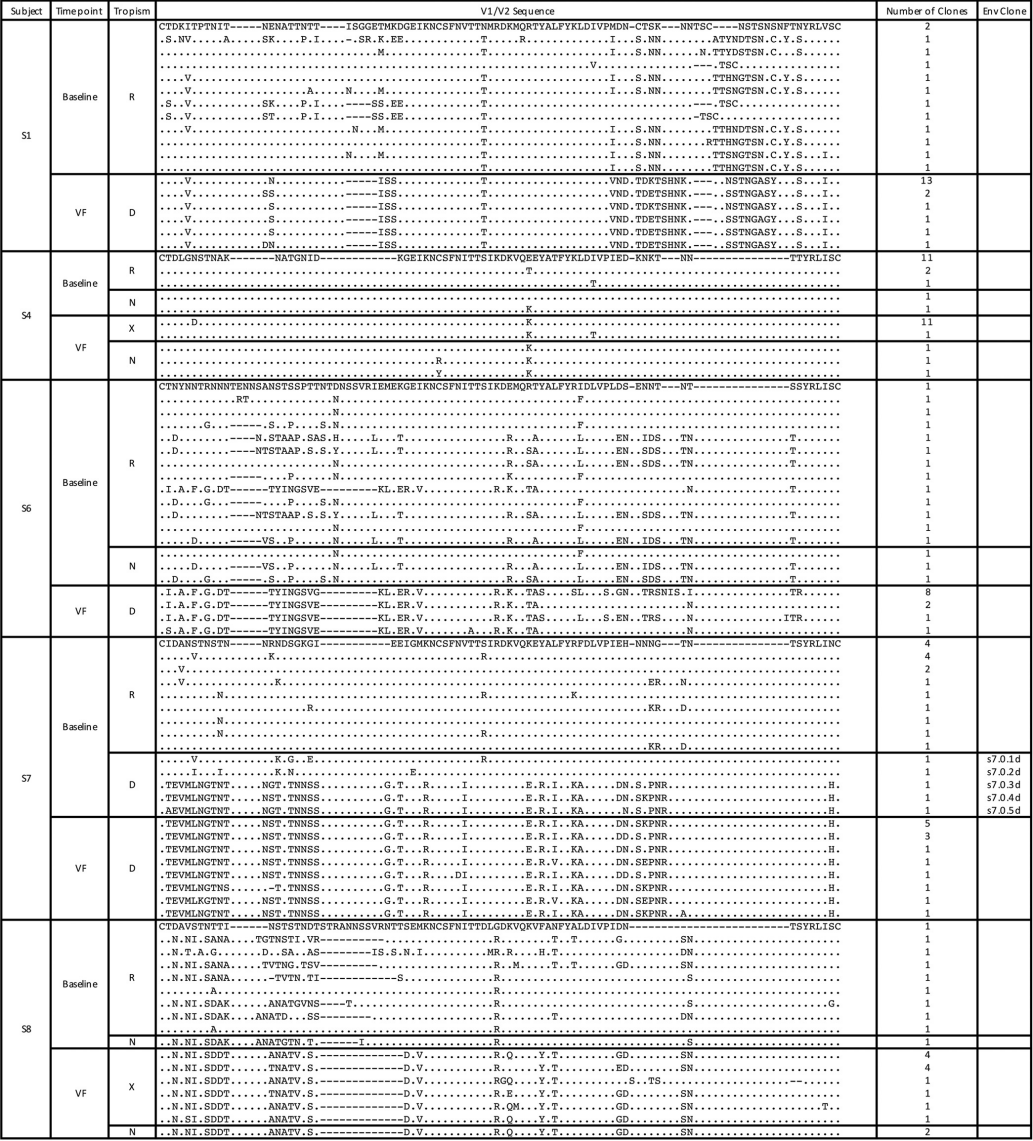
Sequence alignments of V3 loops. Amino acid sequences are shown for R5 (R), X4 (X), dual-tropic (D), or nonfunctional (N) clones, as well as the number of times the identical sequence was obtained, for each subject at baseline and at time of VF. Charges for the V3 loops are also shown, as are consensus V3 sequences for clades A to H HIV-1 strains.

V3 amino acid sequence alignments are shown in Fig. 4. The predominant baseline clone V3 amino acid sequence is shown for each subject, and amino acid differences in variants are indicated below for other baseline clones and those obtained from the same subject at VF. Identical sequences are represented by dots. The V3 loop charge, number of clones, and the experimentally determined tropism is shown for each variant. Residues 11 and 25 are boxed, since these were previously identified as key tropism determinants, a critical determinant of the Geno2pheno tropism prediction program (39). In 4 of 5 subjects (subjects 1, 4, 6, and 8), a significant increase in V3 loop charge was noted (*P* = 7.9 × 10^−29^) (Fig. 4). These sequences match the consensus clade B V3 sequence, including the GPGR V3 apex sequence found in clade B sequences rather than the GPGQ V3 apex sequence found in HIV-1 strains of other clades (40). Although most nonfunctional clones exhibited sequences identical to those of functional clones, two baseline clones from subject 4 showed highly atypical sequences (with a Y-to-C substitution at V3 residue 21 or a GLGR V3 apex).

Expected tropism was determined by inputting the V3 sequence into Geno2pheno. The predicted tropism of each clone was then compared with the actual tropism obtained from the functional tests. Notably, subject 7 showed one clone with an inconsistency between the predicted and actual tropism: S7.b.4d (Fig. 4). When inputted into Geno2pheno, this clone had a false-positive rate of 20.7% and was predicted to be sensitive to CCR5 antagonists. In contrast, our phenotypic test found S7.b.4d to be functionally dual-tropic. Since the functional assays were replicated and repeated with at least two independent experiments, it is unlikely that the inconsistency was due to experimental errors. Furthermore, individual alignments of V3 domains of Env sequences from subject 7 showed that multiple clones with the same V3 domain were R5- and dual-tropic (Fig. 4). Taken together, these findings suggest that domains outside V3 may affect tropism, and inclusion of those regions may improve the predictive accuracy of genotypic algorithms.

Phylogenetic trees for other domains of the envelope protein were also constructed (see Fig. 3 and Fig. S1 in the supplemental material). Significant differences were identified in the phylogenetic trees between R5 and non-R5 clones in the V1/V2, V4, and C3 domains for four of five subjects. Notably, only the V1/V2 phylogenetic analysis correctly placed S7.b.4d among the cluster of X4-using clones.

Alignments of individual V1/V2 sequences were analyzed in an attempt to identify any common mutations or patterns. Notably, V1/V2 sequences were less diverse and more homogenous to one another following treatment (Fig. 4). These results are consistent with our previous study (38) and suggest that the V1/V2 domains may play a role in coreceptor switching under a CCR5 antagonist. However, further analysis of the alignments showed no shared mutations. Sequences were also analyzed for increases or decreases in sequence length, total charge, and N-glycosylation (41, 42). No consistent pattern was identified. These results provide further evidence that V1/V2 mutations may be patient-specific.

**FIG 5:**
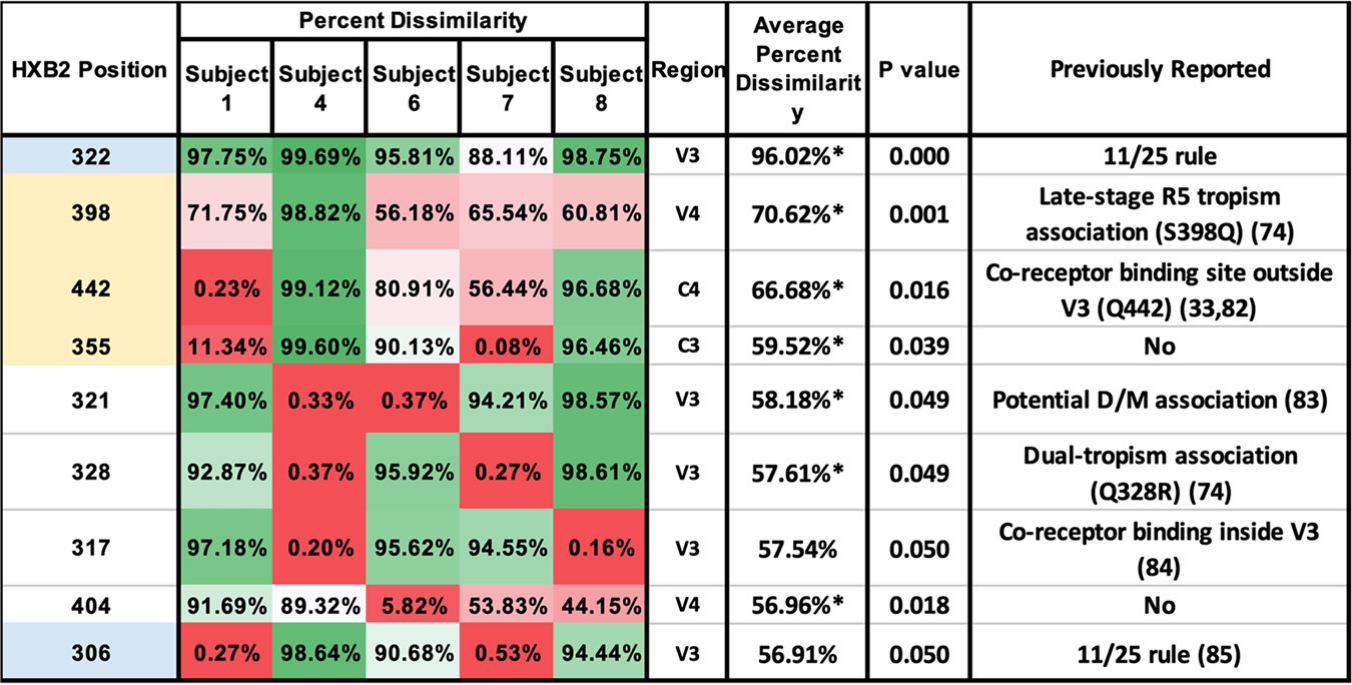
Percent dissimilarity for specific envelope residues that differed most frequently between baseline and VF libraries. Sites were ranked by highest average dissimilarity calculated from the five subjects. Dissimilarity values are denoted using a color scale, with the highest percent dissimilarity indicated as dark green and the lowest as dark red. The position of each residue, as determined by alignment with the HXB2 sequence is shown, as well as its regional location within envelope. Sites 306 and 322 correspond to those identified by the 11/25 rule and are highlighted in blue. Other sites with consistent changes in amino acid characteristics are highlighted in yellow. The average percentage dissimilarity is also shown for all five subjects. The *P* value is indicated for the average percent dissimilarity of that specific site among all patients compared to the average dissimilarity of all sites among all patients. The figure also lists whether or not these specific residues were noted previously (33, 74, 82–85).

Sequence alignments, based on NGS data, show the 9 positions of greatest SNP divergence in each subject (Fig. 5). These positions map to residues within the V3, V4, C3, and C4 domains. Positions 306 and 322 map to residues 11 and 25 in the V3 domain, respectively. At position 398, the residue within the V4 domain of baseline sequences was frequently replaced with a glycine in the VF sequences. At position 442 within the C4 domain, a charged residue in baseline sequences was frequently replaced with an uncharged residue in VF sequences. At position 355 within the C3 domain, a substitution with an uncharged residue in VF sequences was frequently identified.

## DISCUSSION

Technological advances have led to new insights into HIV-1 phylogeny (43). Single-genome amplification of subgenomic and full-length genomic HIV-1 sequences was developed to preserve linkage among polymorphisms in the same viral genome copy, to limit impact from PCR-induced misincorporation and recombination or bacterial selection during cloning. Although most studies have used standard Sanger sequencing for analysis, our previous study and a couple of others sequenced Illumina- or PacBio-based pools of single-genome amplicons (33, 44–46). Recently, Pacific Biosciences SMRT and Oxford MinION Nanopore sequencing were applied for longer reads (47). The current study utilized both single-genome Sanger sequencing analysis and Illumina NGS to characterize HIV-1 quasispecies.

The current study and several prior studies suggested that X4-using HIV-1 variants arise from preexisting minority quasispecies during CCR5 antagonist treatment (46, 48, 49). This was predicted by mathematical modeling approaches (50). Our findings highlight the importance of mutants present at frequencies of 1% or less in the development of drug resistance. This indicates the advantage of deep-sequencing technology for genotypic studies. Subject 7 highlights the failure to identify preexisting dual-tropic clones in the baseline quasispecies. Thus, when evaluating a patient’s eligibility for CCR5 antagonist therapy, combining phenotypic and genotypic analyses may provide a more accurate representation of the population tropism.

In the absence of CCR5 antagonists, a minority of individuals exhibit major HIV-1 population shifts from CCR5 to CXCR4 use (51). Depletion of memory CD4^+^ T cells that coexpress CCR5 and CXCR4 may select for X4-tropic strains, which could increase the range of target cells to include naive CD4^+^ T cells (18, 52–57). With CCR5 antagonists, extreme and rapid HIV-1 quasispecies shifts are seen (34). In this study, we took advantage of the selective pressure from VCV to analyze the genetic development of coreceptor switching.

Previous studies of HIV-1 coreceptor utilization have relied on genotypic or phenotypic assays, but few investigations combined these approaches, as in the current study. Genotypic algorithms, including Geno2Pheno, WebPSSM, PhenoSeq, SCOTH, CoRSeqV3-C, and THETA, were developed to predict coreceptor use of clade B and non-B HIV-1 subtypes (39, 58). However, these approaches may either underestimate or overestimate X4 tropism, depending upon the particular assay and the context in which it was used (59–61).

A surprising finding from the current study was the high proportion of envelope proteins with little or no detectable activity. It is unlikely that these proteins recognize alternative coreceptors without also utilizing CCR5 or CXCR4 (62). The lack of function is likely attributed to limitations in experimental conditions rather than their Env sequence. While some sequences did show that there were significant deletions from the Env gene that could have impaired the activity of the virus, most did not show amino acid sequences that were significantly different from their functional counterparts on the phylogenetic trees. The lack of function may be attributed to sequence changes in the vector backbone that could have disabled the production of infectious virus. Monomeric, nonfunctional envelope proteins have been described on the surface of virions (63). These may have arisen in some cases due to incomplete signal peptide cleavage or partial processing of the gp120/gp41 cleavage site. It is possible that nonfunctional forms of envelope on the virion surface could account for virus capture by nonneutralizing antibodies.

A strength of our study is the longitudinal analysis of a cohort of patients. In a study of patients failing MVC, the dominant route of escape was the emergence of X4-tropic virus (64, 65). When MVC therapy was suspended and the virus was no longer forced to use CXCR4, the major HIV variant in the circulation returned to a solely R5-tropic form (48). Because selective CXCR4 antagonists are not available, there is no way to assess the impact of specific inhibition of X4-tropic virus *in vivo*.

X4 tropism correlates with V3 loop charge, particularly with respect to residues 11 and 25 (66). Other compensatory mutations within other SU and TM domains mediate coreceptor switch (55, 66–73). Our study identified several additional residues within and outside the V3 loop that may be important for coreceptor shift. Sites 398 in V4 and 442 in C4 were previously reported to have been associated with coreceptor shift. The 398Q mutation was found to be commonly associated with end-stage R5 strains and dual-tropic strains (74). These findings suggest that natural coreceptor shift begins with viruses that can utilize CCR5 efficiently. In contrast, our studies indicate a 398G mutation in only dual-tropic strains. We did not find any common 398 mutation in drug-resistant R5-tropic strains from the patients in our earlier study of subjects 2 and 5 (38). Our findings suggest that coreceptor shift in CCR5 antagonist-treated patients may require a different mechanism than natural coreceptor shift. Because CCR5 antagonist therapy acts as an additional selective pressure, coreceptor shift in VCV-treated patients may need to occur more rapidly than coreceptor shift in untreated patients during natural infection. The need for rapid evolution may explain why we observe the bypass of residue 398 mutations in late-stage R5 clones and the differences in the encoded amino acid, glutamine versus glycine.

Zhang et al. previously identified site 442 in the C4 region to be associated with coreceptor shift but did not indicate the exact SNP (33). Our study, which supports the previous study with a single-clone analysis, found that site 442 commonly switched from a charged to uncharged residue. These findings suggest that combining both deep-sequencing methods with single-clone analysis may offer a more comprehensive genetic analysis than NGS alone.

**FIG 6:**
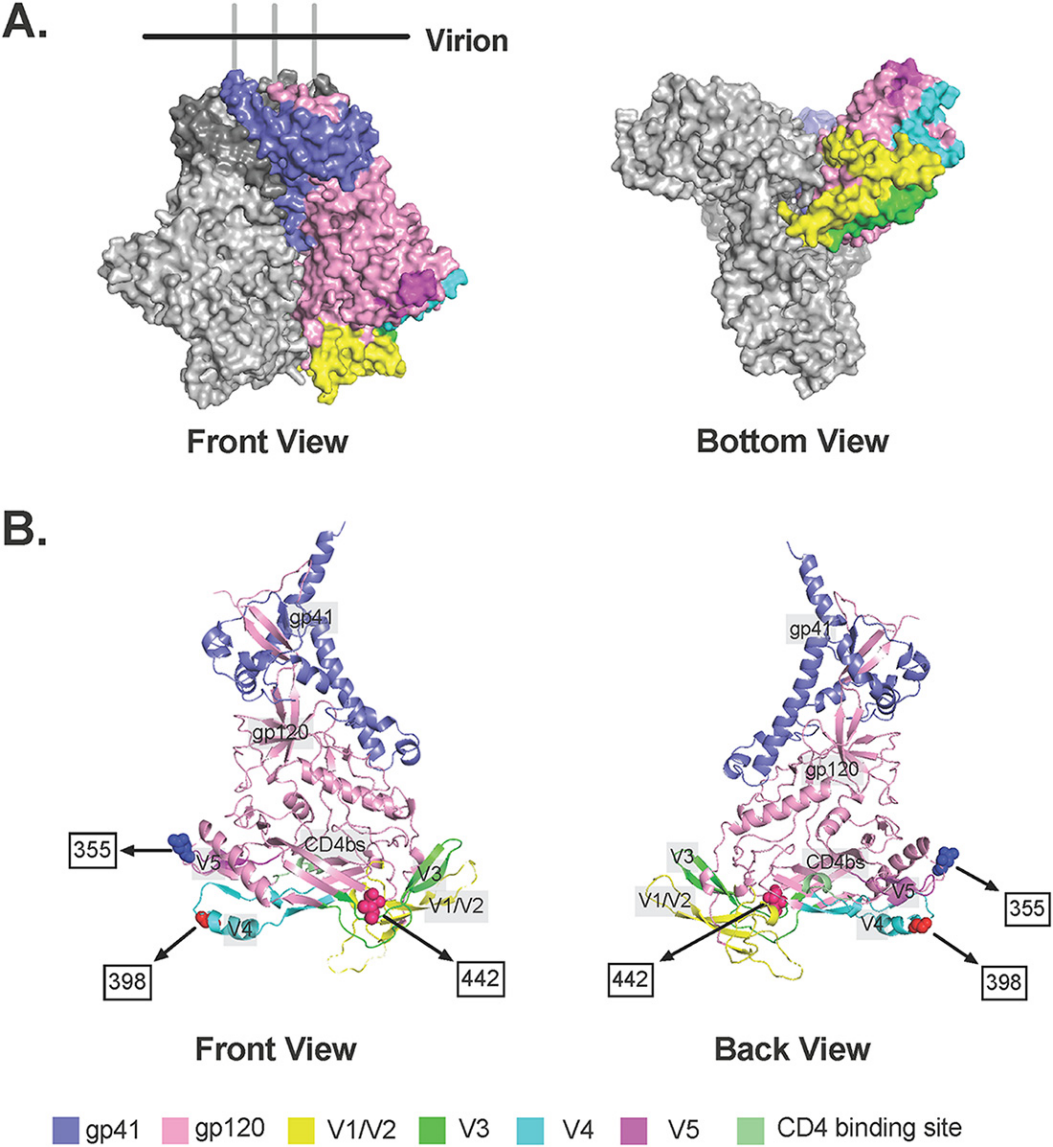
Localization in the envelope protein of key residues influencing coreceptor utilization. A figure derived from the X-ray crystallographic structure of envelope (PDB ID: 6B0N) is shown, along with the positions of the residues with the highest percent dissimilarity. The positions of the V3 and V1/2 loops are also shown.

The positions of the polymorphic residues within the envelope protein, identified from these analyses, are shown in Fig. 6. A representation of the Env trimer (PDB ID: 4zmj) is shown in Fig. 6A with gp41, gp120, and all variable regions highlighted in colors in one of the subunits. Positions of residues identified in NGS analysis of Env are visualized in Fig. 6B, where the sequence was obtained from subject 1 and aligned to a previously identified HIV Env structure (PDB ID: 6B0N) to build the crystal structure (75). Mutations featured in Fig. 6B were not included in Fig. 6A, because the sequence was not sufficiently long, and therefore the positions were not highlighted. As mentioned above, position 442 resides in C4 but is shown on the crystal structure (Fig. 6B) to be neighboring the V3 loop, while position 398 resides in V4. Position 355 is within the C3 domain, but it is not in close proximity to any variable domain or the CD4 binding site. All three positions are located on the bottom of the Env protein (Fig. 6B), and therefore they are likely to encounter the host cell. In addition, these mutations are all found in regions where significant differences were identified between baseline and VF clones in the phylogenetic analysis. These findings taken together suggest that these substitutions may play a role in coreceptor tropism.

Despite a significant change occurring in the V1/V2 domains of four out of five subjects, no shared mutations were identified. This may indicate that V1/V2 mutations are patient-specific. Due to the hypervariability of V1/V2 domains, future studies should focus on identifying patterns rather than individual mutations. Understanding the relationship between the V3 and V1/V2 regions may allow us to better identify any potential motifs. Pastore et al. suggested that V1/V2 mutations offer a gain of function and can compensate for the loss-of-fitness V3 mutations usually required for coreceptor switching (71).

HIV variants that use CXCR4 are less sensitive to V1/V2 and V3 loop-binding broadly neutralizing antibodies, compared to R5-tropic strains (76). This was attributed to a protrusion in the V3 loop or interference with V3 loop exposure by the V1 loop (77). Thus, a detailed understanding of the dynamics of envelope-coreceptor interactions should provide insights into more effective therapies and neutralizing antibodies to block HIV-1 entry.

In summary, we used phylogenetic and functional analyses with NGS and single-clone Env sequence assays of five patients who underwent VCV therapy and experienced a shift in coreceptor tropism. Phylogenetic analysis identified several regions outside V3 that may contribute to their shift in coreceptor usage. Three amino acid substitutions from these regions were identified from NGS and single-clone analysis. These findings may aid in improving the accuracy of current coreceptor prediction algorithms and further the understanding of the interaction between viral Env and host receptors. Future directions should include site-directed mutagenesis to confirm the contribution of the mutations to coreceptor usage identified in this study.

## MATERIALS AND METHODS

### Patient samples.

HIV-1 Env amplicons were obtained from the peripheral blood of six treatment-experienced individuals with clade B HIV-1 who underwent a shift in coreceptor usage, from R5 tropism to X4 or dual tropism, based on the Monogram Bioscience Trofile assay. Samples were collected from two time points: baseline (week 0) and time of VF. VF was defined as an HIV-1 RNA level of <1 log_10_ copies/mL below the baseline level at or after 16 weeks (28).

### Single-clone analysis.

Patient Env plasmid libraries were generated as previously described (33, 38). Env amplicons were cloned into a pNL4-3.Luc.ΔE backbone with an AfeI site. The vector and insert were ligated using NEBuilder master mix with an insert-to-vector molar ratio of 1:1. The ligation products were transformed into ElectroMax Stbl4-competent cells, and positive colonies were screened using the following oligonucleotides: EnvF-ATG (5′-AGAGCAGAAGACAGTGGCAAT-3′) and Nef-EnvR (5′-CCACTTGCCACCCATCTTAT-3′).

### Cell lines.

HEK 293T cells were maintained in complete Dulbecco’s modified Eagle’s medium (DMEM) with 10% fetal bovine serum (FBS), 2 mM l-glutamine, 1 mM sodium pyruvate, 100 U/mL penicillin, and 1× antibacterial-antimycotic solution (100 μg/mL streptomycin and 250 ng/mL amphotericin). U87.CD4 cells were stably transfected with pBABE-CCR5-GFP or pBABE-CXCR4-GFP, constructed as previously described (33), and maintained in DMEM supplemented with 15% fetal bovine serum, 2 mM l-glutamine, 1 mM sodium pyruvate, 1× antibacterial-antimycotic solution, 0.2 mg/mL G418, and 1 μg/mL puromycin.

### Tropism assay.

Tropism of recombinant viruses was determined through a single-round infection, cell-based assay as previously described (38). Three days prior to infection, viral stocks were generated by transfecting 3 μg of viral recombinant plasmid into 1 × 10^6^ HEK 293T cells using TransIT-KT1 transfection reagent (Mirus). Viral stocks were harvested 60 h after transfection and passed through a 0.45-μm filter. One day prior to infection, 50 μL containing 0.3 × 10^6^ cells/mL (U87.CD4.CCR5 or U87.CD4.CXCR4 in 15% FBS–complete DMEM) was plated in a 96-well plate. Following 1 h of incubation with 50 μL of 16 μg/mL DEAE-dextran, the cells were inoculated in duplicate with 50 μL of untitered viral stock. After 48 h, cells were lysed with 0.2% Triton X-100 (Sigma-Aldrich, St. Louis, MO) in phosphate-buffered saline (PBS), and luciferase activity was determined on a GloMax 96 microplate luminometer (Promega, Madison, WI). A positive result was defined as an RLU readout greater than 2 standard deviations above the mean for mock-infected control wells. Each recombinant virus was assayed in duplicate.

### Phylogenic and statistical analyses.

Functional Env isolates identified by the tropism assay were sequenced. Phylogenic analysis was conducted for the full-length Env, each variable (V1 to V5) and constant (C1 to C5) region, and gp41. Amino acid sequences were aligned using MUSCLE (78). Hierarchical clustering analysis was performed using SeaView (79). Phylogenetic trees, apart from those for subject 8, were constructed by using PhyML (80) with the HIV-Wm substitution model, and the reliability of branching order was tested by bootstrap analysis using 1,000 replicates. Due to the low number of sequences collected, subject 8 phylogenetic trees were generated using a distance-based model, BioNJ, bootstrapped with 1,000 replicates (81). Branches with bootstrap values above 95% were considered statistically significant.

### Next-generation sequencing.

DNA samples were sequenced using the Illumina HiSeq 2000 platform, as previously described (33). For functional libraries containing full-length functionally active envelope isolates, Pacific Biosciences (PacBio) RS II platform was also used to identify mutational linkages that are more than 100 bp apart. All sequencing was performed at the McDonnell Genome Institute at Washington University in St. Louis, MO.

For Illumina HiSeq 2000 sequencing, a minimum of 100 ng of DNA sample was processed with the KAPA LTP library prep kit (KAPA Biosystems) to generate and amplify the dual-indexed paired-end ligation library (8 independent 50-μL reaction mixtures, 10 PCR cycles). The end products were fractionated using the DNA 750 chip on the LabChip XT (Perkin Elmer) and purified using AMPure XP beads, and concentrations were determined using quantitative PCR according to the manufacturer’s protocol (Kapa Biosystems). The library preparations were combined in equal molar ratios and loaded on 1 sequencing lane (2 × 101 bp recipe), per the manufacturer’s recommendations (Illumina).

For PacBio RSII sequencing, 750 ng of full-length functional library envelope PCR product of approximately 3 kb in length was used for each sample, using the SMRTBell template preparation kit (Pacific Biosciences). Each sample was run on a single SMRT cell on the PacBio RSII platform (P6v2/C4 chemistry; 240-min movie length).

### Crystal structures.

An Env sequence from subject 1 was aligned using Phyre 2.0 (PDB ID: 6B0N) and visualized in PyMOL (75). The ribbon structure was used for the entire sequence except for residues identified in the NGS, where spheres were used. A surface representation of the Env trimer was generated using PDB ID: 4zmj.

### Statistics.

Frequencies of residues at each position were compared by one-tailed, unpaired *t* tests.

### Data availability.

The sequences identified in this study were deposited with GenBank and assigned accession numbers OM022688 to OM022826.
