# Supplementary material for: Evolution of Multiple Domains of the HIV-1 Envelope Glycoprotein during Coreceptor Switch with CCR5 Antagonist Therapy
Source: Microbiol Spectr. 2022 Jun 21;10(4):e00725-22. doi: 10.1128/spectrum.00725-22 (PMC9431240; doi:10.1128/spectrum.00725-22)
Supplement: Supplemental file 1 — Fig. S1. Download spectrum.00725-22-s0001.pdf, PDF file, 0.7 MB [file spectrum.00725-22-s0001.pdf]

Supplemental Figure Legend:

FIG S1. Phylogenetic trees of A) V5, B) C1, C) C2, D) C4, E) C5, and F) gp41, Results of the hierarchical analysis of variants from functional libraries based on full-length amino acid sequences are shown for each subject. Branch points with bootstrap values greater than or equal to 95% are labeled. The symbols are identical to those used in Fig 2, with open symbols for baseline clones, closed symbols for those obtained at VF, with squares for R5 clones, circles for dual-tropic clones, and triangles for X4 clones. Designations for specific outlier clones are also provided.

Fig. S1

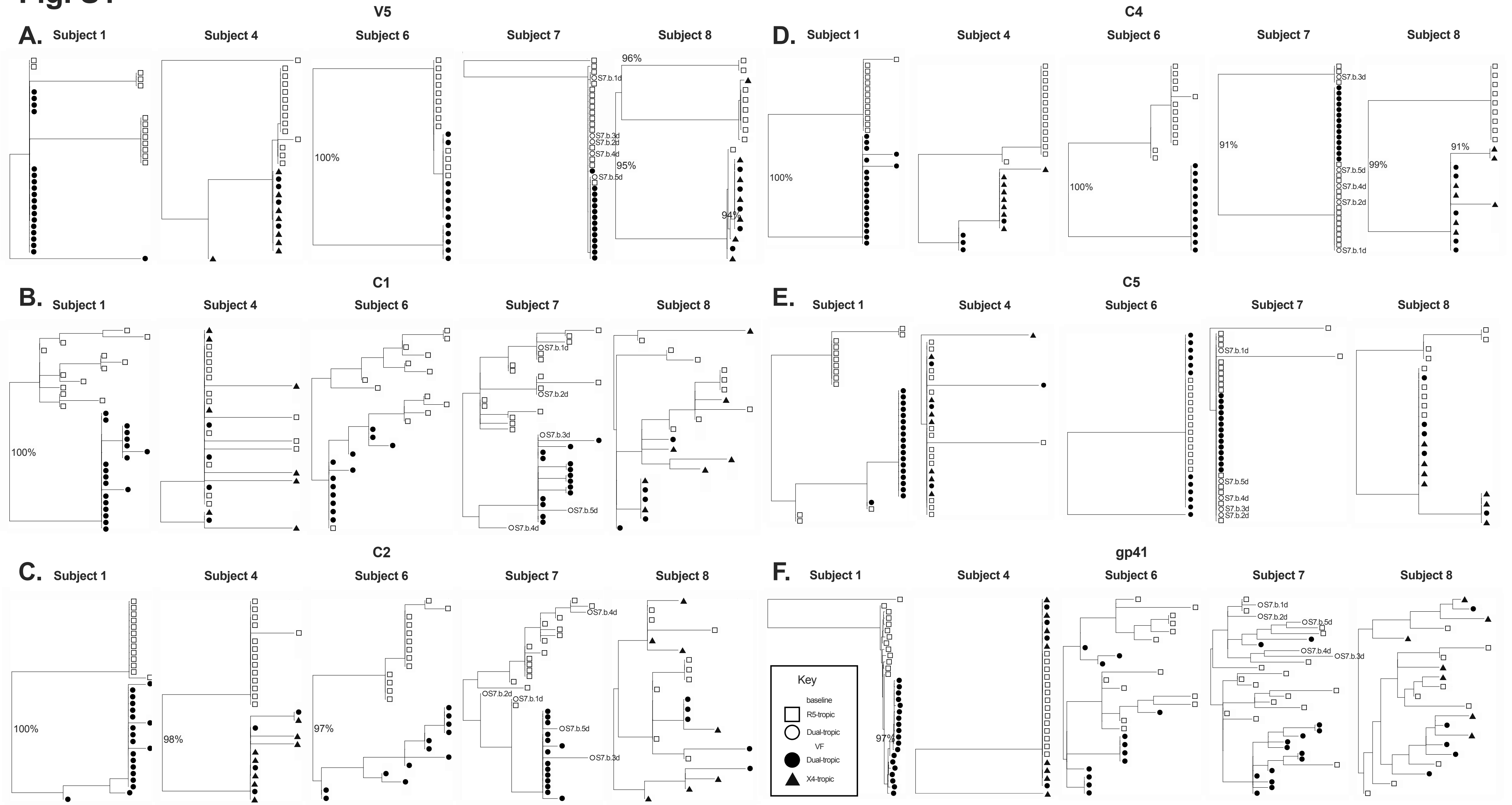
V5
A.
Subject 1
Subject 4
Subject 6
Subject 7
Subject 8
100%
S7.b.1d
S7.b.3d
S7.b.2d
S7.b.4d
S7.b.5d
96%
95%
94%
C4
D.
Subject 1
Subject 4
Subject 6
Subject 7
Subject 8
100%
100%
91%
S7.b.3d
S7.b.5d
S7.b.4d
S7.b.2d
S7.b.1d
99%
91%
C1
B.
Subject 1
Subject 4
Subject 6
Subject 7
Subject 8
100%
S7.b.1d
S7.b.2d
S7.b.3d
S7.b.5d
S7.b.4d
C5
E.
Subject 1
Subject 4
Subject 6
Subject 7
Subject 8
S7.b.1d
S7.b.5d
S7.b.4d
S7.b.3d
S7.b.2d
C2
C.
Subject 1
Subject 4
Subject 6
Subject 7
Subject 8
100%
98%
97%
S7.b.4d
S7.b.2d
S7.b.1d
S7.b.5d
S7.b.3d
gp41
F.
Subject 1
Subject 4
Subject 6
Subject 7
Subject 8
97%
S7.b.1d
S7.b.2d
S7.b.5d
S7.b.4d
S7.b.3d
Key
baseline
R5-tropic
Dual-tropic
VF
Dual-tropic
X4-tropic
